# Epidemiology of SARS-CoV-2

**DOI:** 10.1007/s15010-020-01531-3

**Published:** 2020-10-08

**Authors:** Bernd Salzberger, Felix Buder, Benedikt Lampl, Boris Ehrenstein, Florian Hitzenbichler, Thomas Holzmann, Barbara Schmidt, Frank Hanses

**Affiliations:** 1 Abt. Krankenhaushygiene Und Infektiologie, Universitätsklinikum Regensburg, Franz-Josef-Strauss-Allee 11, 93053 Regensburg, Germany; 2 Gesundheitsamt Regensburg, Sachgebiet Infektionsschutz Und Hygiene, Regensburg, Germany; 3 Fachklinikum Bad Abbach, Klinik für Rheumatologie Und Klinische Immunologie, Regensburg, Germany; 4 Institut für Medizinische Mikrobiologie Und Hygiene, Universitätsklinikum Regensburg, Regensburg, Germany

**Keywords:** SARS-CoV-2, COVID-19, Pandemic, Epidemiology, Basic reproduction number, Incubation period, Mortality, Infection fatality risk

## Abstract

**Purpose:**

SARS-CoV-2 is a recently emerged ß-coronavirus. Here we present the current knowledge on its epidemiologic features.

**Methods:**

Non-systematic review.

**Results:**

SARS-CoV-2 replicates in the upper and lower respiratory tract. It is mainly transmitted by droplets and aerosols from asymptomatic and symptomatic infected subjects. The consensus estimate for the basis reproduction number (R_0_) is between 2 and 3, and the median incubation period is 5.7 (range 2–14) days. Similar to SARS and MERS, superspreading events have been reported, the dispersion parameter (kappa) is estimated at 0.1. Most infections are uncomplicated, and 5–10% of patients are hospitalized, mainly due to pneumonia with severe inflammation. Complications are respiratory and multiorgan failure; risk factors for complicated disease are higher age, hypertension, diabetes, chronic cardiovascular, chronic pulmonary disease and immunodeficiency. Nosocomial and infections in medical personnel have been reported. Drastic reductions of social contacts have been implemented in many countries with outbreaks of SARS-CoV-2, leading to rapid reductions. Most interventions have used bundles, but which of the measures have been more or less effective is still unknown. The current estimate for the infection’s fatality rate is 0.5–1%. Using current models of age-dependent infection fatality rates, upper and lower limits for the attack rate in Germany can be estimated between 0.4 and 1.6%, lower than in most European countries.

**Conclusions:**

Despite a rapid worldwide spread, attack rates have been low in most regions, demonstrating the efficacy of control measures.

Human infections with SARS-CoV-2 were first reported in late 2019, the syndrome was named Coronavirus-Disease-19 (COVID-19). Infections spread rapidly worldwide, in March 2020 WHO declared COVID as a new pandemic. Here we summarize the current knowledge regarding epidemiologic features and parameters of SARS-CoV-2 in a nonsystematic review. Numbers of infections and mortality rates were updated until end of August 2020 [1, 2]. A first epidemic wave could be observed in several countries, giving the opportunity to estimate attack rates of the first wave for some countries.

## Transmission

SARS-CoV 2 replicates mainly in the upper and lower respiratory tract. Replication has also been detected in the GI tract, viral RNA can be present in peripheral blood in severely ill patients [3]. Transmission occurs mainly through respiratory droplets and aerosols [4]. Transmission by other routes has not been convincingly demonstrated in contrast to SARS [5, 6].

One factor for the high infectivity of SARS-CoV2 is its replication in the upper respiratory tract. In contrast to SARS, SARS-CoV-2 can also be transmitted by asymptomatic infected individuals [7, 8].

## Basic reproduction number R_0_, incubation period and superspreading

The basic reproduction number R_0_ can be estimated by observation of infection chains, clusters of infection or by spread in a population. The current consensus estimate for R_0_ for SARS-CoV-2 is between 2 and 3, higher estimates (up to 14.8) have been reported from single outbreaks namely the “Diamond Princess cruise ship [9–11].

The heterogeneity of estimates of R_0_ can best be explained by the high interindividual variance of the likelihood of transmission from a single case. SARS-CoV-2 has shown very effective transmission in several large clusters. In the first outbreak in Wuhan (mainly in healthcare settings) was reported. In contrast, the household infection rates were low [4]. So called superspreading events have, meanwhile, also been reported outside of health care settings, e.g., in religious gatherings [12].

This variance can be described by the overdispersion parameter k (kappa). The lower k, the higher is the variance of interindividual transmission rates (Table 1). Using current estimates of R_0_ (2.5) and kappa (0.1) 60% of infections will not be transmitted, while 10% of infections are responsible for > 80% of transmissions [11].

**Table 1:** Epidemiologic parameters of SARS-CoV-2

Parameter	Value
Basic reproduction number R_0_	2–3 (Consensus) Range: 1.7–14.8
Dispersion coefficient (kappa)	0.1 (0.05–0.2)
Incubation period	Median 5.7 d, 99% of infections within 2–14 d
Serial interval	4.0

Currently the conditions leading to superspreading are not fully characterized, especially the role of biological (e.g., viral load, loud voice) or social factors (e.g., number of contacts).

It is obvious that prevention of superspreading events will have a large impact on transmission.

The median incubation period is 5.7 days, 99% of all infections happen between day 2 and day 14 [13]. The latent period is probably 1 day shorter [13]. The median serial interval of infections has initially been estimated as high as 7.6 days, later observations put this parameter closer to 4 days (Table 1) [10, 14, 15].

## Age distribution of cases

**Fig. 1:**
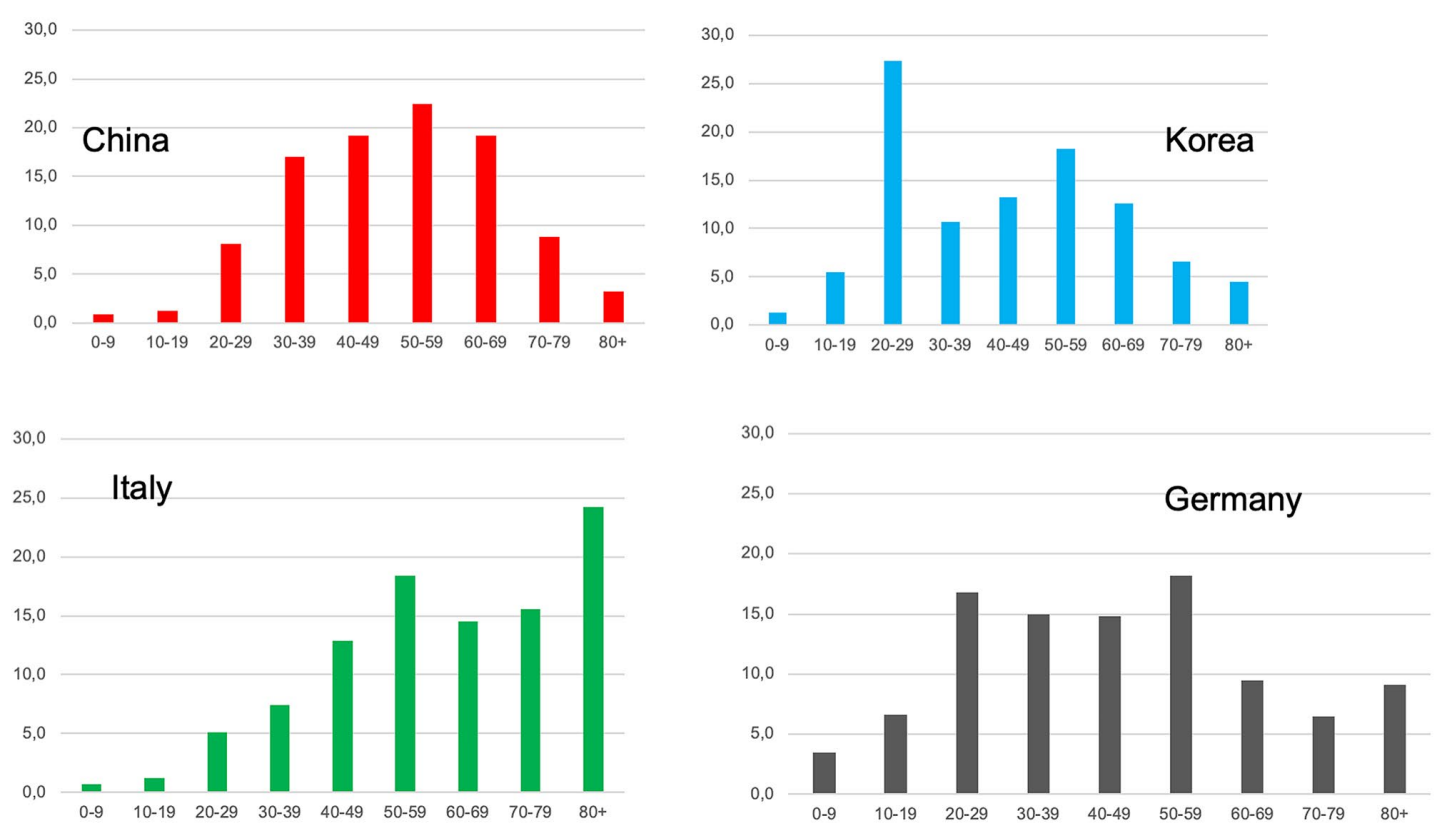
Age distribution of COVID-19 cases in China, Korea, Italy and Germany

**Fig. 2:**
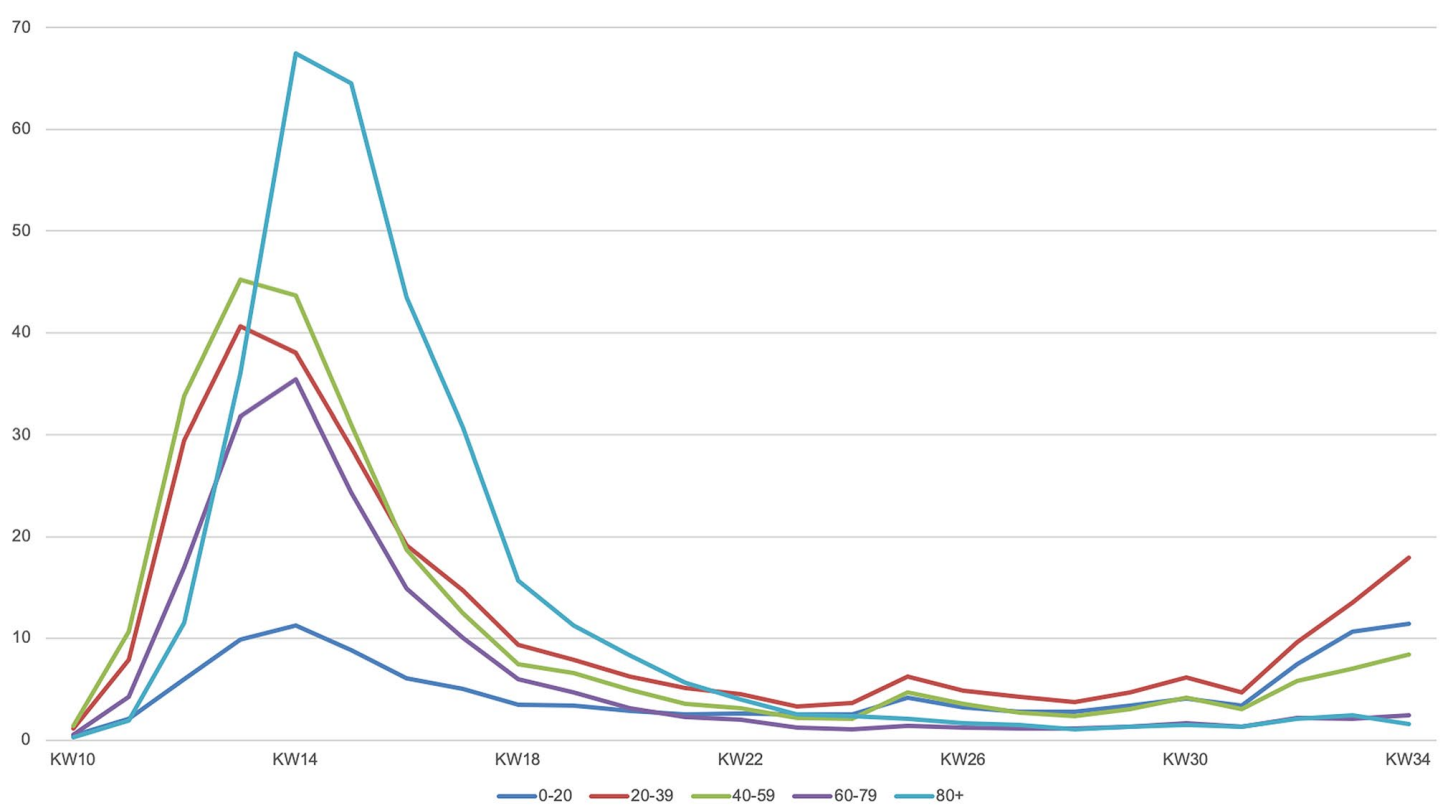
Age specific weekly incidence (cases/100,000) of COVID 19 in Germany, week 10–34/2020

The number of cases in most countries is highest in the age group between 20 and 59 years. Outbreaks in China, Korea, Italy and Germany show different patterns (Fig. 1). In all outbreaks the numbers of infected children (age 0–9) have been small (Fig. 1) [2, 16–18]. In Germany the age specific incidence rates over the first wave of the epidemic show the highest initial incidence in the age group over 80 years and higher incidences in younger people in later stages of the epidemic (Fig. 2) [2].

## Nosocomial and transmission in health care settings

Transmissions in health care settings, both nosocomial and to health care personnel have first been reported in the early period of the Wuhan outbreak. In most settings infection in the index patients was unknown and transmission was associated with emergency procedures, e.g., intubation [4].

Later in the outbreak most infections in health care workers were not classified as health care associated but community transmission. The rate of infected health care personnel in all cases was 2.7% in China, 11% in Italy and 5.8% in Germany [2, 4, 18]. In seroepidemiologic studies from Italy and Spain the seroprevalence of SARS-CoV-2 infection was approximately twice the rate of workers in other occupations (Italy 5.3% vs. 2.8%, Spain 10.2% vs. 4.8%, UK 11.7% vs. 5.3%) [19-21]. Fortunately, the number and rate of severe cases and complications in contrast are low in this group [2, 4, 18].

## Clinical spectrum, severity of disease and long-term outcome

Infection with SARS-CoV has a broad clinical spectrum from asymptomatic, oligosymptomatic to moderate or even severe disease with multiorgan failure.

The rate of asymptomatic infections has been estimated from intensive follow up in regional outbreaks and retrospectively in seroprevalence studies with rates of asymptomic infections ranging from 27 to 40% [20–22]. Overall, approximately 90% of infections are uncomplicated, oligosymptomatic or with moderate symptoms not leading to hospitalization.

Higher age, hypertension, chronic cardiac or pulmonary disease, and immunosuppression are risk factors for severe disease. Rates of hospitalization range in different populations between 4 and 7%. 25% of hospitalized patients require intensive care with a high rate of organ replacement therapy (75% invasive ventilation, 25% renal replacement therapy) [23-26].

Long term outcome, especially with severe lung disease and multiorgan failure is unknown, studies to determine the patterns of sequelae are ongoing.

## How deadly is SARS-CoV-2?

One crucial parameter to determine the severity of a pandemic is the infection fatality rate IFR). Due to the high rate of uncomplicated infections symptomatic cases do not reflect the total number of infections. Thus, the case fatality rate (CFR) will be much higher than the IFR. In COVID-19, CFRs in different countries differ considerably, mainly due to testing strategies and the age distribution of the national population (Tables 2, 3).

**Table 2:** Age specific case-fatality rates in different nations and models for infection fatality rates

Age (years)	Case-fatality rates					Infection-fatality rate (IFR) Modell			
	China	Italy	Germany	Spain	Südkorea*	Indiana, USA**	ENE, Spain	International (Imperial College)	France
0–9	0%	0.1%	0.01%	0.3%	0%	n.d	< 0.01%	0.0016%	0.001%
10–19	0.2%	0%	0.01%	0.1%	0%	0.01%	< 0.01%	0.0069%	0.001%
20–29	0.2%	0.1%	0.03%	0.3%	0%	0.01%	0.01%	0.031%	0.005%
30–39	0.2%	0.3%	0.07%	0.3	0.1%	0.01%	0.025%	0.084%	0.02%
40–49	0.4%	0.9%	0.2%	0.6%	0.2%	0.12%	0.07%	0.16%	0.05%
50–59	1.3%	2.7%	0.8%	1.5%	0.5%	0.12%	0.29%	0.59%	0.2%
60–69	3.6%	10.8%	4.0%	5.2%	1.4%	0.12%	1.15%	1.93%	0.7%
70–79	8.0%	26.6%	13.5%	14.6%	6.7%	0.12%	3.38%	4.28%	1.9%
80+	14.8%	34.6%	26.4%	21.8%	21.0%	?**	8.12%	7.8%	8.3%
Overallt	2.3%^#^	13.8%	3.8%	8.2%	1.6%	0.26%**	0.83%	0.657%	0.5%**

*Case-fatality-rate, by intensive contact tracking probably close to IFR

**Only community-dwelling people, institutionalized excluded

^#^Estimated with 90% still hospitalized, later corrected to 5.2%

**Table 3:** SARS-CoV-2 infections, seroprevalence and attack rates in different countries

Country population	China (only Hubei)	Spain	Italy	Germany	France (only French model appl [25])	United Kingdom
Population (millions)	57.20	46.94	60.36	83.02	66.99	66.65
Seroprevalence						
National	4%	5.4%	2.5%	n.d	n.d	6.0%
Regions	Hubei 4% Hongkong 0%	Madrid 11.5% Barcelona 6.8% Baleares 1.1% Asturia 1.4%	Lombardy 7.5% Piemont 3.0% Tuscany 1.0% Sicily 0.3%	Bad Feilnbach 6% Gangelt 15.5%	n.d	London 13.0% Yorkshire 3.9% South-West 2.8%
Est. Cases and attack rates of SARS-CoV-2						
Cases notified (8/2020)	68,053	250,273	260,307	233,776	223,419	316,371
Overall CFR	5.2%	7.5%	13.7%	4.0%	13.6%	14.3%
Cases—lower bound est	0.19 Mio	0.74 Mio	0.96 Mio	0.34 Mio	2.1 Mio	1.75 Mio
Cases—upper bound est	0.96 Mio	3.18 Mio	4.46 Mio	1.49 Mio	6.0 Mio	8.85 Mio
Attack rate (min–max)	0.33–1.68%	1.57–6.78%	1.51–7.39%	0.42–1,80%	3.3–9.3%	2.6–13.1%

Investigators from the Imperial College, London, have first established a mathematical model to estimate age dependent case- and infection fatality rates from a high number of cases (Table 2). Other estimates have been based on related models, cohort studies, on national seroprevalence studies and outbreaks, which have probably been monitored almost completely, e.g., South Korea (Table 2) [17, 19, 24, 27, 28].

There is yet no single consenset estimate for the IFR, but most models calculate this parameter between 0.5 and 1 (Table 2) The two models with the lowest values exclude especially a group with a high fatality rate, institutionalized persons above the age of 80 [24, 27].

IFRs have not been calculated for the most severe Influenza pandemics in the twentieth century (1918, 1957, 1968), a direct comparison is thus not possible. Case fatality rates for pandemics between 1918 have been estimated by a group from the CDC. The CFR of the Influenza 1918 pandemic is highest with 2.04, followed by 0.1–0.3 for Influenza 1957 and up to 0.05 for Influenza 1968 [29].

All current estimates for the CFR of COVID-19 (except in countries with probably incomplete mortality data) are higher than the CFR for Influenza 1918. In addition, organ replacement therapy had not been established widely in the time of any of these pandemics. Taking the 25% mortality rate of COVID-19 patients requiring invasive ventilation, for a direct comparison of CFRs the COVID-19 CFR would have to be multiplied by an appropriate factor (e.g., 4).

But the severity of a pandemic depends not solely on the IFR. Severity is also dependent on the age distribution of fatalities and the attack rate. In sharp contrast to COVID-19, a high mortality rate in younger patients (20–49 years) has been reported from Influenza 1918 and the attack rate with symptomatic cases was between 9 and 40%, higher than for almost all national COVID-outbreaks so far (Table 3) [29].

**Fig. 3:**
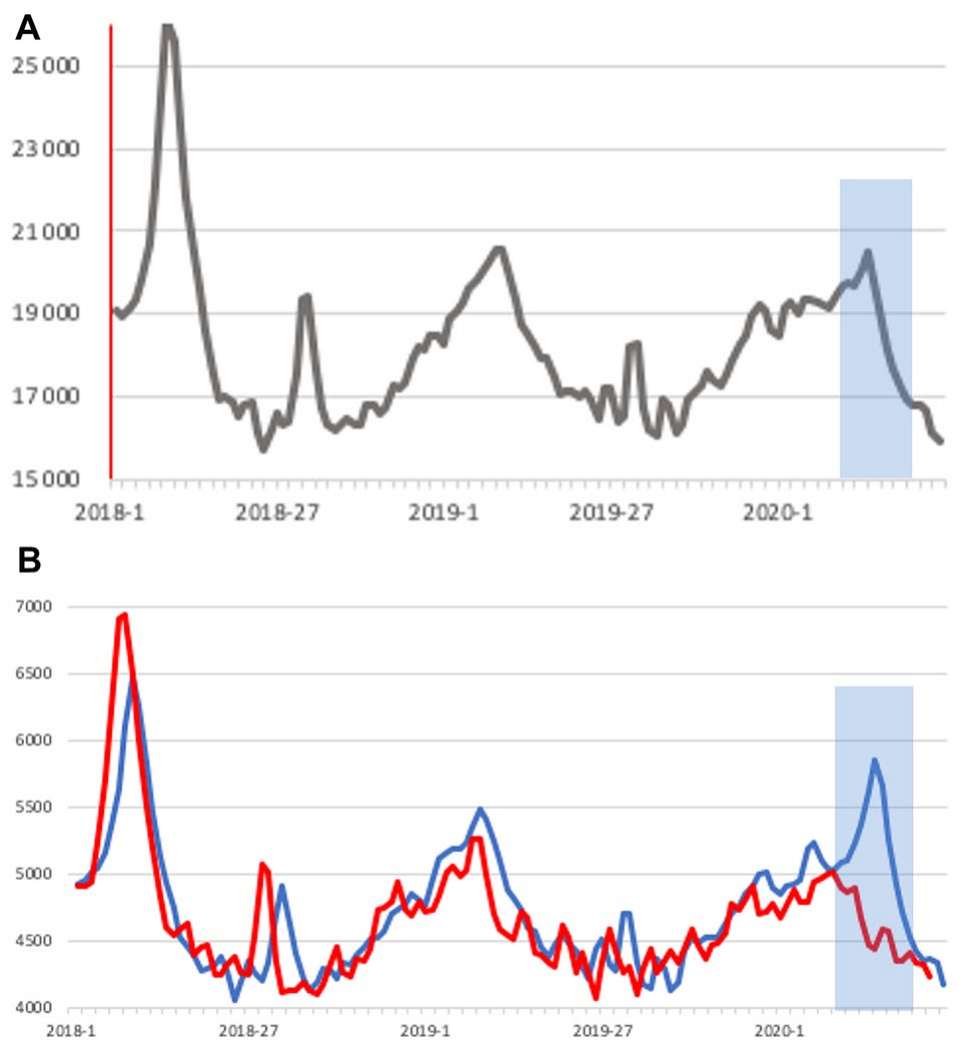
**a**, **b** Weekly deaths 2018-5/2020 in **a** Germany and two regions, **b** Bavaria plus Baden-Württemberg (combined, blue line) (**c**) Mecklenburg-Vorpommern plus Saxonia-Anhalt combined, red line), adjusted for population size by factor 4.5 (weekly deaths in means over 2 weeks. Period with deaths due to COVID shaded in gray (weeks 8–20/20,209)

Severity of an epidemic can also be measured by excess mortality. Excess mortality due to COVID-19 has been reported from a number of regions, e.g., 24 nations from Europe with 185,000 excess deaths in the first 18 weeks of 2020 [30]. No excess mortality, but a signal of COVID mortality can be seen in German weekly deaths, a week signal for Germany (Fig. 3a), a pronounced signal for the two states with the highest incidence (Fig. 3b) and no signal for the two states with the lowest incidence of SARS-CoV-2 (Fig. 3b) [31].

## Spread of SARS-CoV-2 and attack rates in the first wave

SARS-CoV-2 has rapidly spread worldwide in the last 9 months. WHO declared COVID-19 as a pandemic in March 2020. Strict control measures have limited outbreaks in some Asian countries, e.g., China, Korea, Vietnam and Thailand, but SARS-CoV-2 outbreaks have been reported in a large number of nations [32]. At the end of April 2020 3 million cases and 200,000 deaths had been reported worldwide, at the end of august 2020 over 25 Mio infections and 800,000 deaths have occurred [1].

National or regional attack rates can be measured by seroprevalence studies or estimated using IFR models (Table 3). Seroprevalence studies from different nations differ widely, with additional variation between regions (Table 3) [19, 33, 34, 12, 20, 21].

Using age dependent case fatality rates and adjustment by age-dependent infection fatality rates, attack rates can be estimated. Using the models of listed in Table 2, we calculated minimum and maximum case numbers in different age categories and summarized them to the upper and lower bounds of infections and attack rates (Table 3).

These estimates are consistent with seropravelence studies and demonstrate, that even in outbreaks leading to overflow of ICU capacities, the actual attack rate has been low (e.g., Lombardy with 7%), lower than for most of the Influenza pandemics. For Germany we estimate the attack rate between 0.4 and 1.6% (Table 3). All attack rates measured or estimated so far are too low to induce a protective herd immunity.

## Control and prevention of COVID-19

Following the outbreak in Wuhan, the province Hubei was isolated completely in February 2020. Citizens were neither able to leave or reach the province. A curfew was installed, infected persons were isolated and close contacts quarantined. Some of these measures were also implemented in other Chinese cities and regions, where smaller outbreaks occurred following the new year festivities in China. At the start of control measures 5000 cases were notified, these rose to 70,000 in March. Afterwards the number of cases dropped dramatically, and since April, only singular cases or small clusters have been reported from China.

Contact restrictions should have an effect on transmission and reduce Rt. With a reduction of Rt below 1, an outbreak can be controlled.

A group from the Imperial College of London has evaluated control measures and lockdowns in several countries in Europe. They have found large effects of these measures, putting an end to a first wave of spreading until May 2020. The effect of single measures in bundle interventions are more difficult to determine and most countries have implemented similar measures. The comparison of the CFRs between COVID-19 and Influenza pandemics is a reminder, that control and prevention measures should be kept at a high level.

## Conclusion

SARS-CoV-2 is a newly emerged coronavirus. Human infections have first been detected in late 2019 in Wuhan, China. In the months following, the virus has rapidly spread worldwide. SARS-CoV-2 replicates mainly in the upper and lower respiratory tract and is highly infectious. Droplets and aerosol are the main routes of transmission and infection occurs also through asymptomatic infected individuals. Nearly 90% of cases are uncomplicated, in a minority of cases severe disease and complications occur. Risk factors for severe disease are older age, hypertension, diabetes mellitus, chronic heart or pulmonary disease, and immunodeficiency.

A first wave of COVID-19 has be reported in most European countries between February and May 2020, leading to high excess mortality. Control measures restricting social contacts, travel activities and commerce have lead to control of outbreaks in many countries with a high efficacy.

The current case-fatality rates in most countries are higher than for known Influenza pandemics, but the fatality rates in younger people and the attack rates in most countries are lower than in the most severe, the Influenza 1918.

## Abbreviations

CFR: Case fatality rate
IFR: Infection fatality rate
R_0_: Basic reproduction number
